# Clinical-Pathological Conference Series from the Medical University of Graz: Case No 174: A 25-year-old pregnant woman from Afghanistan with headache and a blast flag in the automated differential blood count

**DOI:** 10.1007/s00508-020-01681-2

**Published:** 2020-06-29

**Authors:** Philipp K. Bauer, Peter Krippl, Elisabeth Fabian, Karoline I. Mayer-Pickel, Robert Krause, Franz Bauer, Guenter J. Krejs

**Affiliations:** 1 Division of Infectious Diseases and Tropical Medicine, Department of Internal Medicine I, Medical University of Vienna, Vienna, Austria; 2 Department of Internal Medicine, Feldbach-Fuerstenfeld Hospital, Feldbach-Fuerstenfeld, Austria; 3 Division of Gastroenterology and Hepatology, Department of Internal Medicine III, Medical University of Vienna, Vienna, Austria; 4 Department of Gynecology and Obstetrics, Medical University of Graz, Graz, Austria; 5 Section of Infectious Diseases and Tropical Medicine, Department of Internal Medicine, Medical University of Graz, Graz, Austria; 6 Division of Hematology, Department of Internal Medicine, Medical University of Graz, Graz, Austria; 7 Division of Gastroenterology and Hepatology, Department of Internal Medicine, Medical University of Graz, Auenbruggerplatz 15, 8036 Graz, Austria

**Keywords:** Tertian malaria, Plasmodium vivax, Chloroquine, Primaquine, Pregnancy

## Presentation of case

### Dr. K.I. Mayer-Pickel:

The patient speaks Pashto. Originally from the Laghman province in Afghanistan, she has been living in Austria for 4 months. Her last menstruation was 4 months ago, now she is pregnant in the 15th week of gestation expecting twins. The patient’s history is positive for one miscarriage; she and her husband are cousins, so they are blood related (consanguinity). Her travel history has been negative since she came to Austria. In the 13th week of gestation, a routine check-up in the obstetrics outpatient clinic of Graz University Medical Center revealed an unremarkable pregnancy with dichorionic diamniotic twins. In the 15th week of gestation, the patient again came to the outpatient clinic because of a cold, headache and cough. She was diagnosed with an upper respiratory tract infection and was given ambroxol, a saline nasal spray and paracetamol as needed. Laboratory data at that time showed: leukocytes 4.9 × 10^9^/L (normal: 4.4–11.3 × 10^9^/L), erythrocytes 3.37 × 10^12^/L (normal: 4.10–5.10 × 10^12^/L), hemoglobin 10.2 g/dL (normal: 12.0–15.3 g/dL), hematocrit 28.4% (normal: 35.0–45.0%), neutrophils 80% (normal: 50–75%), lymphocytes 13% (normal: 20–40%), platelets 196 × 10^9^/L (normal: 140–440 × 10^9^/L), C‑reactive protein (CRP) 28.9 mg/L (normal: <5.0 mg/L) and fibrinogen 516 mg/dL (normal: 210–400 mg/dL). In case of persistent complaints the patient was advised to come to the emergency room (ER) of Graz University Medical Center. Two weeks later, the patient presented in the ER with a retrosternal pressure sensation radiating into the left arm and back, a 2-week history of headache, fever (39.9 °C), and poor appetite for 2 days. The patient showed tachycardia with a heart rate of 129 bpm. Echocardiography and Duplex sonography of the leg veins were unremarkable. Laboratory data: leukocytes 3.9 × 10^9^/L, erythrocytes 2.94 × 10^12^/L, hemoglobin 8.6 g/dL, hematocrit 25.1%, CRP 35.7 mg/L, lactate dehydrogenase (LDH) 261 U/L (normal: 120–240 U/L) and ferritin 233 ng/mL (normal: 30–150 ng/mL). The condition worsened 3 days later and the patient complained of headache but no fever, and laboratory data revealed pancytopenia and markedly elevated CRP. She was admitted to the Department of Obstetrics of the Graz University Medical Center. Laboratory data on admission: CRP 61.3 mg/L, leukocytes 2.87 × 10^9^/L, erythrocytes 2.15 × 10^12^/L, hemoglobin 6.6 g/dL, LDH 330 U/L, neutrophilic granulocytes 52%, lymphocytes 41%, myelocytes 1% (blast warning), platelets 102 × 10^9^/L. The condition of both fetuses was good. Abdominal sonography revealed a 16-cm spleen. Because of severe anemia, two units of packed red blood cells were administered. The consulting neurologist could not identify a specific cause for the patient’s persistent headache. According to the hematologist, there was no evidence of acute leukemia or leukemic lymphoma. No fragmentocytes were seen on a blood smear.

A diagnostic test was performed.

## Differential diagnosis

### Dr. P. Krippl:

This is a very interesting and complex case. Let me begin by summarizing the key facts. The patient under discussion is a 25-year-old pregnant woman originally from Afghanistan (Laghman province). Except for one miscarriage, her medical history is unremarkable. Recently, she had an upper respiratory infection with markedly increased CRP and decreased leukocytes. Furthermore, her hemoglobin level was slightly decreased; however, anemia is frequently found in pregnant women. She presented in the ER 2 weeks later and complained of pressure behind her breast bone, radiating into the left arm and back, a headache lasting for 2 weeks and fever (39.9 °C) as well as tachycardia. At that time, laboratory data showed a progressive increase in CRP, and a further drop in leukocytes and hemoglobin. Three days later, her condition worsened. On admission, the patient presented with a headache, but no fever. Laboratory data revealed pancytopenia, a further increase in CRP, a hemoglobin level of only 6.6 g/dL and elevated LDH. There was a blast flag in the automated differential blood count. The blood smear showed one myelocyte, but was negative for fragmentocytes, and there were no findings suggesting a hematologic malignancy. Abdominal sonography revealed a slightly increased spleen (16 cm).

In summary, the patient developed pancytopenia between the 13th and 18th weeks of gestation. During this period there was a significant drop in hemoglobin from 10.2 to 6.6 g/dL, accompanied by an increase in LDH suggesting hemolytic anemia. Detection of one myelocyte in the automated differential blood count does not seem to be a key finding in this case because these cells may occur sporadically during severe infections. Fragmentocytes, which are associated with conditions such as valvular heart disease, mechanical heart valve, hemolytic uremic syndrome (HUS), thrombotic thrombocytopenic purpura (TTP), disseminated intravascular coagulopathy (DIC), thrombosis and march hemoglobinuria [1] were not found in this patient. However, laboratory data revealed a progressive increase in CRP levels during the 13th and 18th weeks of gestation. Thus, the leading features in this case are hemolytic anemia and infection.

Hemolysis means the destruction or removal of erythrocytes from the circulation before the end of their normal life span of 120 days and may present as acute or chronic anemia, reticulocytosis or jaundice. Hemolysis can be caused by two different pathophysiological mechanisms: (1) intravascular hemolysis, which is the destruction of erythrocytes in the circulation with the release of cell contents into plasma, and (2) extravascular hemolysis, which is the removal and destruction of erythrocytes with membrane alterations by the macrophages of the spleen and liver [2]. The causes of hemolytic anemia are often categorized as hereditary (Table 1) or acquired (Table 2). Hereditary corpuscular forms of anemia are due to inborn defects of one of three main components of erythrocytes: enzymes, cell membrane or hemoglobin. The most common enzymopathy resulting in hemolysis is glucose-6-phosphate dehydrogenase (G6PD) deficiency, which is an X‑linked disorder that primarily affects men. It afflicts over 400 million people worldwide [4]. Of the many G6PD variants existing, only a few cause hemolysis [5]; however, with the Mediterranean variant, severe hemolysis may occur [6, 7]. The G6PD is a critical enzyme in the production of glutathione, which protects erythrocytes against oxidative stress. In most patients, clinical and laboratory manifestations reflect episodic hemolysis triggered by infection, specific drugs or favism, which consequently leads to oxidation and denaturation of hemoglobin, and formation of Heinz bodies by intracellular precipitation. Heinz bodies are removed by the spleen, leaving erythrocytes with a missing section of the cytoplasm; these “bite cells” can be seen in a stained blood smear. The altered erythrocytes undergo both intravascular and extravascular destruction [2].

Among membranopathies causing hemolysis, hereditary spherocytosis, an autosomal dominant disorder caused by mutations in the erythrocyte membrane skeleton protein genes, should be mentioned. Due to a defective protein structure, the membrane undergoes a progressive deterioration in structure resulting in spherocytes, which can be identified on a peripheral blood smear. Since these cells are unable to pass through the splenic cords, they are degraded and ingested by the monocyte-macrophage system. Usually, hereditary spherocytosis is manifested as a chronically compensated, mild to moderate hemolytic anemia [2].

Chronic hemolysis is also characteristic of diseases with a disturbed hemoglobin synthesis, i.e. hemoglobinopathies, such as sickle cell anemia and thalassemias. The prevalence of hemoglobinopathies varies widely across different regions of the world. While it is between 0.5 and 1% in central Europe, it reaches up to 30% in some African regions and up to 60% in some Arab countries [8], which is the result of parasite-induced selection due to reduced susceptibility to severe malaria [9]. Normal adult hemoglobin (HbA) is designated αA_2_βA_2_; variant hemoglobin is derived from gene abnormalities affecting the alpha-globin genes (HBA1 or HBA2) or beta-globin structural genes [10, 11]. Today, more than 1000 hemoglobin variants (e.g. amino acid insertions, deletions or mutations in the intervening sequences (introns)) have been identified [11]. Sickle cell anemia is caused by a point mutation leading to a substitution of valine for glutamic acid in the 6th position of the beta chain of hemoglobin. Sickling and oxidative stress due to hemoglobin S membrane abnormalities and an overall impaired deformability of sickle cells result in splenic removal of cells and, to some degree, in intravascular hemolysis. Thalassemias are inherited multifactorial microcytic hypochromic anemias, characterized by defects in the synthesis of the alpha or beta subunit of the hemoglobin tetramer. In severe forms of alpha thalassemia (hemoglobin H disease) and beta thalassemia (intermedia and major), deficiency of one globin chain results in intracellular precipitation of the excess chain, which in turn leads to damage of the cell membrane and hemolysis [2].

**Table 1:** Causes of hereditary hemolytic anemias (adapted from [2, 3])

Corpuscular	*Enzymopathies*
	Glucose-6-phosphate dehydrogenase deficiency
	Enzymopathies of the glycolytic pathway (pyruvate kinase deficiency, glucose phosphate isomerase deficiency, hexokinase deficiency, aldolase deficiency, phosphofructokinase deficiency, etc.)
	Enzyme defects of the nucleotide metabolism (e.g. pyrimidine 5’-nucleotidase deficiency, adenylate kinase deficiency)
	*Membranopathies*
	Hereditary spherocytosis
	Hereditary elliptocytosis
	Hereditary pyropoikilocytosis
	Hereditary stomatocytosis
	*Hemoglobinopathies*
	Sickle cell anemia
	Thalassemia
Extracorpuscular	Familial (atypical) hemolytic uremic syndrome
	Upshaw-Schulman syndrome (the recessively inherited form of thrombotic thrombocytopenic purpura)

**Table 2:** Causes of acquired hemolytic anemias (adapted from [2, 3])

Corpuscular	Paroxysmal nocturnal hemoglobinuria
	Hemoglobinopathy caused by carboxyhemoglobin
Extracorpuscular	*Entrapment*
	*Immune*
	Autoimmune hemolytic anemia
	Warm-reactive IgG antibody
	Cold-reactive IgM antibody (cold agglutinin disease)
	Cold-reactive IgG antibody (paroxysmal cold hemoglobinuria)
	Alloimmune hemolytic anemia
	Drug-induced hemolytic anemia
	Autoimmune
	Haptene
	*Infection*
	*Traumatic*
	Impact hemolysis
	Macrovascular defects—prostheses
	Microvascular causes (TTP, HUS, DIC, HELLP syndrome)
	*Due to toxic effects on the membrane*
	Spur cell anemia
	External toxins (e.g. animal or spider bites, metals such as copper and lead, drugs)
	Zieve’s syndrome in alcoholic liver disease

*TTP* thrombotic thrombocytopenic purpura, *HUS* hemolytic uremic syndrome, *DIC* disseminated intravascular coagulopathy, *HELLP* hemolysis, elevated liver enzymes, low platelet count, *Ig* immunoglobulin

Besides hereditary diseases causing corpuscular damage and subsequent hemolysis, also relevant and worthy of note are hereditary diseases that lead to hemolysis via an extracorpuscular pathomechanism, namely familial (atypical) HUS and the Upshaw-Schulman syndrome (the recessively inherited form of TTP). However, due to a negative family history and the absence of clinical features, the diagnosis of such an inherited disease seems unlikely in the discussed patient.

Various differential diagnoses have to be considered regarding acquired hemolytic anemia (Table 2). For example, paroxysmal nocturnal hemoglobinuria is a rare acquired erythrocyte membrane abnormality leading to hemolytic anemia. Besides the laboratory findings of hemolytic anemia, reticulocytosis, increased LDH, hemoglobinuria and hemosiderinuria, most patients clinically present with fatigue, malaise and lethargy. In some patients, thrombosis or bone marrow failure syndrome may be found as well [12]. The different causes of extracorpuscular acquired hemolytic anemia such as (auto)immune-associated hemolytic anemia, macroangiopathies or microangiopathies, intoxication and others (Table 2) can all be ruled out in our patient due to lacking clinical presentation or significant laboratory findings including a characteristic peripheral blood smear. Considering the laboratory findings and clinical presentation with signs of infection in the discussed patient, her hemolytic anemia is most likely due to infection. Indeed, various infectious diseases are associated with hemolytic anemia (Table 3). However, because of a lack of clinical features of infection with most pathogens listed in Table 3, only infections with selected pathogens such as *Babesia divergens, Leishmania donovani, Plasmodium falciparum, Plasmodium malariae, Plasmodium vivax, *Coxsackie virus, Epstein-Barr virus (EBV) or cytomegalovirus (CMV) are possible causes in this case. Infections with Coxsackie virus, EBV or CMV are quite common and also frequently associated with mild to moderate hemolysis [2]. Thus, they should be considered in patients with signs of infection and hemolytic anemia; however, due to the lack of clinical symptoms and hints in the medical history, these viral infections seem unlikely in the discussed patient.

**Table 3:** Infectious pathogens causing hemolytic anemia (in alphabetic order [13])

Aspergillus sp.	Leptospira sp.
*Babesia microti*	*Mycoplasma pneumoniae*
*Babesia divergens*	*Neisseria meningitidis*
*Bartonella bacilliformis*	
*Campylobacter jejuni*	*Plasmodium falciparum*
*Clostridium welchii*	*Plasmodium malariae*
Coxsackie virus	*Plasmodium vivax*
Cytomegalovirus	Rubella virus
*Streptococcus pneumoniae*	Rubeola virus
Epstein-Barr virus	Salmonella sp.
*Escherichia coli*	Shigella sp.
*Haemophilus influenzae*	*Mycobacterium tuberculosis*
Hepatitis A virus	*Toxoplasma gondii*
Herpes simplex virus	Trypanosoma sp.
Human immunodeficiency virus	Varicella virus
Influenza A virus	*Vibrio cholerae*
*Leishmania donovani*	*Yersinia enterocolitica*

The genus *Babesia* comprises more than 100 species of protozoan pathogens that infect erythrocytes of many vertebrate hosts [14]. Babesiosis shows a worldwide distribution and affects a wide variety of mammalian species [15]. However, it was only in the last 30 years that some species of *Babesia* were recognized as important pathogens in humans. In Europe, fatal babesiosis due to infection with *Babesia divergens* was first described in 1956 in the former Yugoslavia [16]. *Babesia divergens* is a natural pathogen of cattle and the main pathogen of human babesiosis in Europe [17–19]. The most important mode of parasite transmission is through the bite by an infected Ixodes tick. *Babesia divergens* has a complex life cycle with asexual and sexual reproduction cycles, which alternate between vertebrate host and tick vector. When *Babesia divergens* sporozoites enter the human host, they immediately target erythrocytes using multiple complex interactions between parasite proteins and the host cell surface, but they do not invade other host cells [15]. Once inside the erythrocytes, the parasite begins a cycle of maturation and growth due to intense intracellular proliferation. The parasite then builds a complex structure of infected erythrocytes by loading different numbers of intracellular parasites. This protects the host and ensures parasite survival from hemolysis that would result from massive invasion [20].

Leishmaniasis is a protozoan parasitic disease found in the tropics, subtropical areas and southern Europe. Leishmania parasites are transmitted by the bite of phlebotomine female sand flies [21]. While *Leishmania tropica* and *Leishmania major* cause cutaneous, mucocutaneous and diffuse cutaneous leishmaniasis, *Leishmania infantum* and *Leishmania donovani* are associated with visceral leishmaniasis (also known as kala-azar or black disease due to a peculiar gray discoloration of the skin of the hands, feet, abdomen and face) [22, 23]. Visceral leishmaniasis, which is a systemic infection of the reticuloendothelial system, is endemic in more than 60 countries, including southern Europe, North Africa, the Middle East, Central and South America and the Indian subcontinent [24]. The parasite comes in two forms: aflagellate or amastigote, and flagellate or promastigote. As an amastigote it exists and proliferates in the mononuclear phagocytic system, especially the spleen, liver and bone marrow, resulting in hepatosplenomegaly and hematological manifestations such as anemia, leukopenia, thrombocytopenia, pancytopenia, histiocytosis or DIC [23]. The cause of anemia in patients with visceral leishmaniasis is multifactorial and includes sequestration and destruction of erythrocytes in the enlarged spleen, immune mechanisms and altered cell membrane permeability. Erythrocyte survival and ferrokinetic studies have suggested that hemolysis is the major cause of anemia (typically normocytic normochromic) in patients with visceral leishmaniasis [25, 26]. Further clinical features of visceral leishmaniasis include persistent fever and a peculiar gray discoloration of the skin of the hands, feet, abdomen and face [23].

Malaria is a protozoan disease transmitted by the bite of infected anopheles mosquitoes. In 2018, an estimated 228 million cases of malaria occurred worldwide with a death toll of 405,000 [27]. There are four species of the genus *Plasmodium* (*Plasmodium falciparum, Plasmodium vivax, Plasmodium ovale, Plasmodium malariae*) that cause nearly all malarial infections in humans [28]. *Plasmodium falciparum *is the most prevalent malarial parasite in the African region (99.7%) and South-East Asia (50%), the eastern Mediterranean region (71%) and the western Pacific region (65%). Globally, 53% of the *Plasmodium vivax *burden is in the South-East Asia region, with the majority being in India (47%) [27]. The first symptoms of malaria are nonspecific such as headache, fatigue, abdominal discomfort and muscle aches, followed by fever (up to 40 °C and above with tachycardia), i.e. they are similar to features of a viral infection. In some patients, a predominance of symptoms such as headache, chest pain, arthralgia or myalgia can be found. Although headache may be severe in malaria, there is no neck stiffness or photophobia. Typical clinical findings described in acute uncomplicated infection include fever, malaise, enlarged spleen and hemolytic anemia due to reduced erythrocyte deformability and consequent acceleration of erythrocyte destruction and removal by the spleen in conjunction with ineffective erythropoiesis. The classical malaria paroxysms characterized by fever spikes and chills occurring at regular intervals are quite unusual and suggest infection with *Plasmodium vivax* or *Plasmodium ovale* [28]. The fact that the discussed patient had migrated from Afghanistan only 4 months prior to presentation, and that 40% of Afghan immigrants with infectious diseases suffer from malaria [29], strongly suggests the diagnosis of malaria. Afghanistan has the world’s third highest malaria burden. About 75% of the population reside in areas at risk of malaria transmission. The highest prevalence of malaria is found in eastern Afghanistan where about 90% of all cases of confirmed *Plasmodium falciparum* and *Plasmodium vivax* infections occurred in only 6 districts (of a total of 400) in 2017. Among these districts is Laghman where the incidence is more than 5 per 1000 population per year [27, 30]. *Plasmodium vivax* affects a larger geographic area of the country than *Plasmodium falciparum* and accounts for 80–95% of malaria cases [30]. Since the discussed patient originates from Laghman, it is highly likely that she suffers from tertian malaria due to infection with *Plasmodium vivax*. For a conclusive diagnosis of malaria, the asexual form of the parasite has to be demonstrated in stained thin and possibly thick peripheral blood smears [28].

## Dr. P. Krippl’s diagnosis

Tertian malaria (*Plasmodium vivax*)

## Discussion of case

### Dr. G.J. Krejs:

The consulted hematologist Dr. F. Bauer will comment on the peripheral blood smear.

### Dr. F. Bauer:

In contrast to the automated blood count, no blasts or other malignant cells could be found on the blood smear of the discussed patient. Indeed, the smear showed severe anemia, and mild leukopenia and thrombocytopenia, but no pathologically altered erythrocytes such as fragmentocytes, sickle cells, target cells or bite cells suggesting a microangiopathy, hemoglobinopathy or enzymopathy. However, the blood smear did reveal erythrocytes with inclusions, which together with the clinical presentation of the patient strongly suggested the diagnosis of malaria.

### Dr. G.J. Krejs:

With a working diagnosis of malaria, we consulted Dr. Krause, head of the Section of Infectious Diseases and Tropical Medicine at this institution.

### Dr. R. Krause:

**Fig. 1:**
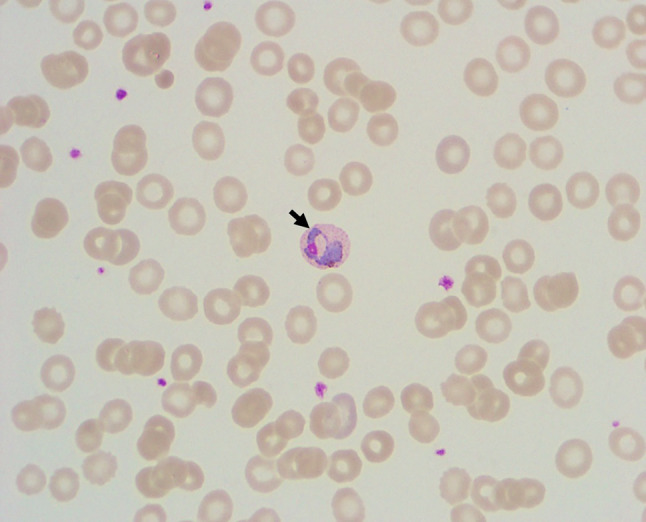
Thin blood smear of the patient with tertian malaria, showing a *Plasmodium vivax* trophozoite within an erythrocyte (*arrow*)

Based on the history of the discussed patient, her origin and the clinical presentation including splenomegaly, anemia, thrombocytopenia and increased LDH, malaria had to be strongly suspected in this case. Afghanistan is a country which is highly endemic for malaria [27, 31, 32]. The disease occurs at altitudes below 2000 m above sea level and is most prevalent in snow-fed river valleys and areas used for growing rice. The highest prevalence of malaria (90% of all cases) is found in the eastern part of the country. Both *Plasmodium falciparum* and *Plasmodium vivax* infections can be found in Afghanistan. Transmission of *Plasmodium vivax *malaria takes place from May/June to November with negligible transmission occurring between December and April; however, many *Plasmodium vivax *infections relapse during the spring season. Transmission of *Plasmodium falciparum* primarily occurs between August and October, but its prevalence is unstable in this region and can fluctuate markedly from year to year due to climatic variations. Based on the infectious reservoir of hypnozoites and the ability of the disease to develop at lower temperatures in the vector, *Plasmodium vivax* affects larger geographic regions of Afghanistan than *Plasmodium falciparum* [30]. The discussed patient originates from Laghman, a district with an incidence of malaria of more than 5 per 1000 population per year [27, 30]. Her blood smear showed *Plasmodium vivax* trophozoites in erythrocytes (Fig. 1), which confirmed the diagnosis of tertian malaria.

*Plasmodium vivax* is geographically the most widely distributed cause of malaria with up to 2.5 billion people at risk and an estimated 80–300 million clinical cases every year, including severe disease and death [33, 34]. The most obvious feature that distinguishes *Plasmodium vivax* from *Plasmodium falciparum* is the development of dormant hypnozoites in the liver that cause relapse due to subsequent spill over into the bloodstream. *Plasmodium vivax* has a complex life cycle in which the parasite undergoes more than ten stages of cellular differentiation and invades at least four types of cells within two different hosts. Once infective sporozoites are inoculated into the human skin by female anopheles mosquitoes, they reach the bloodstream and enter hepatocytes, where they develop into either an actively dividing schizont (releasing merozoites into the bloodstream) or a dormant stage called hypnozoite [35]. Activation of hypnozoites weeks, months or years later causes reactivation of the infection, clinical malaria and the potential for transmission of the sexual gametocyte forms. During the erythrocytic stages, *Plasmodium vivax* merozoites predominantly invade reticulocytes [36, 37] leading to enlarged and more deformable cells [38]. All blood stage forms of *Plasmodium vivax* are found in the peripheral circulation. Enhanced deformability might somehow help infected cells to safely pass through the spleen. Further, the parasite also has adhesive knobs for sequestration in deep vascular beds as a way to avoid passage through the spleen [39]. Recent data showed that late asexual blood stage *Plasmodium vivax* parasites are capable of cytoadhering due to endothelial host receptors [40, 41]. In addition, data indicate that *Plasmodium vivax* parasites expand and replicate outside of the circulation in the hematopoietic niche of the bone marrow and possibly the spleen, affecting bone marrow function and host immunity [42]. However, this host-parasite interaction and its implication for diagnosis and treatment has to be further elucidated.

For the treatment of acute tertian malaria, chloroquine is available and should be administered in multiple doses within 48 h (initial dose 1000–1250 mg, after 6 h 500–750 mg, and 500 mg after 24 and 48 h), also in pregnant women [43]. In patients with *Plasmodium vivax* or *Plasmodium ovale* infection, several additional attacks (relapses) after months or even years without symptoms may occur because both *Plasmodium vivax* and *ovale* have dormant liver stage parasites (hypnozoites) that may reactivate. To reduce the chance of such relapses, treatment with 15 mg primaquine for 14 days should follow the treatment of the first attack. However, during pregnancy, disease suppression with 500 mg chloroquine once a week instead of with primaquine should follow the initial treatment; treatment of hypnozoites should be postponed to the postpartum period [43].

### Dr. K.I. Mayer-Pickel:

Treatment with chloroquine was well-tolerated by the discussed patient. Her pregnancy with dichorionic diamniotic twins was unremarkable. In the 28th week of gestation, a mild discordance in the growth of both fetuses was observed on abdominal sonography. During the further course of the pregnancy, a minor ventricular septal defect was found in both fetuses. Vaginal delivery was initiated in the 38th week of gestation and two girls (body weight 2820 g, APGAR 9/10/10 and body weight 2750 g, APGAR 9/10/10) were born without any complications. Due to their ventricular septal defects, regular visits in the outpatient heart valve clinic were advised for the children.

### Dr. G.J. Krejs:

In the 33-year history of 174 clinical-pathological conferences from the Medical University of Graz, this is the second case of malaria.

### Dr. R. Krause:

Following the delivery, the patient was advised to take primaquine, but obviously did not adhere to her doctors’ instructions and 4 weeks later, she came back to the ER with fever. Recurrence of tertian malaria was diagnosed. She was again treated with chloroquine, followed by a course of primaquine. This time she adhered to the treatment recommendations and subsequently remained free of disease.

## Final diagnosis

Tertian malaria (*Plasmodium vivax*)
